# Monoacylglycerol Lipase (MAGL) Inhibition Attenuates Acute Lung Injury in Mice

**DOI:** 10.1371/journal.pone.0077706

**Published:** 2013-10-25

**Authors:** Carolina Costola-de-Souza, Alison Ribeiro, Viviane Ferraz-de-Paula, Atilio Sersun Calefi, Thiago Pinheiro Arrais Aloia, João Antonio Gimenes-Júnior, Vinicius Izidio de Almeida, Milena Lobão Pinheiro, João Palermo-Neto

**Affiliations:** 1 Neuroimmunomodulation research group, Department of Pathology, School of Veterinary Medicine, University of São Paulo, São Paulo, Brazil; 2 Laboratory of Experimental Toxicology, Department of Clinical and Toxicological Analyses, School of Pharmaceutical Sciences, University of São Paulo, São Paulo, Brazil; 3 Department of Pharmacology, Institute of Biomedical Sciences, University of Sao Paulo, São Paulo, Brazil; University Hospital Freiburg, Germany

## Abstract

Endocannabinoid signaling is terminated by enzymatic hydrolysis, a process that, for 2-Arachidonoylglycerol (2-AG), is mediated by monoacylglycerol lipase (MAGL). The piperidine carbamate, 4-​nitrophenyl- ​4-​(dibenzo[d] [1,3]dioxol-​5-​yl (hydroxy) methyl) piperidine- 1-​carboxylate (JZL184), is a drug that inhibits MAGL and presents high potency and selectivity. Thus, JZL184 increases the levels of 2-AG, an endocannabinoid that acts on the CB_1_ and CB_2_ cannabinoid receptors. Here, we investigated the effects of MAGL inhibition, with a single dose (16 mg/kg, intraperitoneally (i.p.)) of JZL184, in a murine model of lipopolysaccharide (LPS) -induced acute lung injury (ALI) 6, 24 and 48 hours after the inflammatory insult. Treatment with JZL184 decreased the leukocyte migration into the lungs as well as the vascular permeability measured through the bronchoalveolar lavage fluid (BAL) and histological analysis. JZL184 also reduced the cytokine and chemokine levels in the BAL and adhesion molecule expression in the blood and BAL. The CB_1_ and CB_2_ receptors were considered involved in the anti-inflammatory effects of JZL184 because the AM281 selective CB_1_ receptor antagonist (1-(2,4-dichlorophenyl)-5-(4-iodophenyl)-4-methyl-*N*-4-morpholinyl-1*H*-pyrazole-3-carboxamide) and the AM630 selective CB_2_ receptor antagonist ([6-​iodo-​2-​methyl-​1-​[2-​(4-​morpholinyl)ethyl]-​1H-​indol-​3-​yl](4-​methoxyphenyl)-​methanone) blocked the anti-inflammatory effects previously described for JZL184. It was concluded that MAGL inhibition, and consequently the increase in 2-AG levels, produced anti-inflammatory effects in a murine model of LPS-induced ALI, a finding that was considered a consequence of the activation of the CB_1_ and CB_2_ receptors.

## Introduction

The discovery of the CB_1_ and CB_2_ cannabinoid receptors and their major ligands N-arachidonoylethanolamide (anandamide) and 2-arachidonoylglycerol (2-AG) led to the characterization of the endocannabinoid system [1][2][3][4]. This system has become a subject of great interest in pharmacology due to its remarkable distribution in mammals and its capacity to play a modulating role in diverse physiological functions including immunomodulation and inflammation [5]. 2-AG and anandamide are regulated by the catabolic enzymes monoacylglycerol lipase (MAGL) [4] and fatty acid amide hydrolase (FAAH) [6], respectively.

To investigate the MAGL role in endocannabinoid metabolism and signaling *in vivo*, Long et al. (2009) [7] described in mice a potent and selective inhibitor for this enzyme termed 4- ​nitrophenyl-​4-​(dibenzo [d] [1,3] dioxol–5–​yl (hydroxy) methyl ) piperidine-​1-​carboxylate (JZL184) that decreases the 2-AG hydrolysis in both the Central Nervous System (CNS) and in the peripheral tissues, including in the lungs [8]. JZL184 has also been shown to have potent immunosuppressive and anti-inflammatory properties in some rodent models of inflammation [9][10]. Employing this drug (16 mg/kg, given systemically), a remarkable variety of CB_1_-dependent effects such as analgesia, hypomotility and hypothermia were shown in mice [7]. Using JZL184 (8 mg/kg i.p.), Busquets-Garcia et al. (2011) [11] highlighted the anxiolytic effects of this drug in mice and suggested that the observed effects might have been induced by 2-AG actions on the CB_2_ receptors; an antinociceptive effect related to the CB_1_ and the CB_2_ receptor activation has also been reported in mice [11].

Acute lung injury (ALI) and its most severe form, acute respiratory distress syndrome (ARDS) in humans, is a lung disease with an acute onset that is characterized by bilateral pulmonary infiltrates with neutrophil accumulation [12][13]. ALI has been shown to result in persistent respiratory failure and prolonged dependence on mechanical ventilation, increased susceptibility to multi-organ dysfunction and mortality [14]. Despite numerous innovations in intensive care medicine, the ALI-related mortality rate has been reported to vary by approximately 40% [14][15]. 

Although the search for the effects of endocannabinoids such as anandamide and 2-AG on immunity has received considerable research attention, the effects of MAGL inhibition on inflammation models are still unclear. This work was designed to investigate the potential anti-inflammatory effect of MAGL inhibition in a murine model of LPS-induced ALI using JZL184 as a pharmacological tool. Elucidating the roles of CB_1_ and CB_2_ cannabinoid receptor activation on JZL184 effects was also an objective of this work.

## Materials and Methods

### Animals

Male C57BL/6 mice from our own colony, weighing 22-28 g and approximately 60 days old, were used. The animals were housed under conditions of controlled temperature (22–26 °C) and artificial light (12-h light/12-h dark, lights on at 7:00 a.m.), with free access to rodent chow and water. The experiments were performed in a different room at the same temperature as the animal colony. The animals were transferred and maintained in their home cages 7 days before the beginning of the experiments. This study protocol was approved by the Bioethical Committee of Care and Use of Laboratory Animal Resource of the School of Veterinary Medicine, University of São Paulo, Brazil (permit number: 2255/2011); the guidelines are similar to the guidelines of the National Institutes of Health (NIH), USA. All surgery was performed under ketamine and xylazine anesthesia, and all efforts were made to minimize suffering.

### Drugs

The JZL184 (piperidine carbamate 4-​nitrophenyl- ​4- ​(dibenzo[d] [1,3]dioxol- 5-​yl (hydroxy) methyl) piperidine-1-​carboxylate) (Cayman Chemical, MI, USA) vehicle was prepared in 15% dimethyl sulfoxide (DMSO), 4.25% Tween 80, 4.25% polyethylene glycol (PEG) 400 and 76.5% saline. The AM281 (1-(2,4-dichlorophenyl)-5-(4-iodophenyl)-4-methyl-*N*-4-morpholinyl-1*H*-pyrazole-3-carboxamide) (Sigma Aldrich, St Louis, USA) vehicle and the AM630 ([6-​iodo-​2-​methyl-​1-​[2-​(4-​morpholinyl)ethyl]-​1H-​indol-​3-​yl](4-​methoxyphenyl)-​methanone) vehicle (Tocris Bioscience, Bristol, UK) were prepared in 10% DMSO, 10% Tween 80 and 80% saline.

### Experimental protocol and group formation

Two experiments were conducted in accordance with the Good Laboratory Practice (GLP) protocols and quality assurance methods. The first experiment was performed to investigate the possible effects of JZL184 on ALI. For that purpose, 61 mice were randomly assigned into four groups: control (C1 and C2) and experimental (E1 and E2) groups. The JZL184 was intraperitoneally (i.p.) administered at a single dose of 16 mg/kg (0.1 mL/10 g weight) to the animals of the experimental groups. The control groups received a similar volume of JZL184 vehicle alone. Sixty minutes after treatment, ALI was induced, as described below. Mice were analyzed 6, 24 and 48 hours after the LPS (C2 and E2 groups) or the saline (C1 and E1 groups) intranasal (i.n.) instillation.

The participation of the CB_1_ or the CB_2_ receptors in the JZL184-induced effects on ALI was assessed in the second experiment, which used 99 mice. As in the first experiment, the animals were divided into two experimental (E1 and E2) and two control (C1 and C2) groups that received i.p. JZL184 (16 mg/kg) or a similar volume of its vehicle (0.1 mL/10 g). Within each of these 4 groups, the animals were again divided into 3 groups that received one of the following treatments i.p. 30 minutes before JZL184 or the JZL184 vehicle: AM281 (2.5 mg/kg), AM630 (2.5 and 5.0 mg/kg) or AM281 and AM630 vehicle (0.1 mL/10 g). The ALI was induced 60 minutes after the JZL184 or JZL184 vehicle treatment, and the mice were analyzed 6, 24 and 48 hours later.

The doses and the treatments were based on the following past literature: for JZL184 [7][8], for AM281 [16] and for AM630 [17][18].

### Acute lung injury (ALI)

ALI was performed according to previous work from our laboratory [13]. The mice were anesthetized with ketamine and xylazine injected i.p. (100 and 10 mg/kg, respectively) before intranasal instillation of LPS. *Escherichia coli* LPS (O55:B5 L2880, Sigma-Aldrich, St. Louis, USA) at a concentration of 100 μg/mL or sterile 0.9% saline were intranasal instilled into the mice (1 μL/g of mouse body weight). Six, twenty-four and forty-eight hours after the induction of inflammation, mice were anesthetized and killed by exsanguination through the inferior vena cava for the bronchoalveolar lavage fluid (BAL), blood and bone marrow collected and analysis.

### Leukocyte trafficking

#### Blood

Blood studies were performed as described by Ligeiro-Oliveira et al. (2004)[19]. The mice were anesthetized as described above, and the samples of the blood were taken from the abdominal vena cava with plastic syringes containing 10 µL of 10% EDTA and subsequently diluted 1:20 in Turk´s fluid (3% acetic acid).The total number of cells present in the blood was counted with the aid of a light microscope in Neubauer chambers. Differential leukocyte counts were performed on smears stained with Rosenfeld’s dye using standard morphological criteria.

#### Bronchoalveolar lavage fluid (BAL)

BAL was performed according to Tavares de Lima, Sirois and Jancar (1992)[20]. After exsanguination, the BAL was collected. The lungs were flushed with 1.5 mL of phosphate-buffered saline (PBS) solution through the use of a cannula inserted by tracheostomy. After collection, the BAL fluid was centrifuged at 250 x g for 5 min. The supernatant was stored, and the remaining cell pellet was resuspended in 1 mL of PBS solution. Total leukocyte counts were performed by adding 10 µL of crystal violet to 90 µL of the cell suspension. Neubauer chambers were used for cell counting under a light microscope. The differential cell count was carried out on the cytocentrifuged (250 x g for 5 min) (FANEM, São Paulo, Brazil) cell suspension (100 µL) stained with Rosenfeld’s dye using standard morphological criteria.

#### Bone marrow

The percentage of granulocytes in the bone marrow was quantified from the femoral marrow lavage fluid that was obtained as described by Ligeiro-Oliveira (2004)[19]. Briefly, mice had their femurs removed and both epiphyses were cut off. A needle connected to a plastic syringe containing PBS (5 mL) was inserted into each femoral marrow to allow cell removal by flushing. The cell suspension was centrifuged at 250 x g for 5 min; the cell pellet obtained was resuspended for the total leukocyte count as describe above for the BAL. To analyze the percentage of granulocytes in the bone marrow, the cells were adjusted to 1 x 10^6^ and were incubated with FITC-conjugated anti-mouse Ly6G, clone 1A8 (Granulocytes, Biolegend, CA, USA), according to the manufacturer’s instructions. A flow cytometer (FACSCalibur, Becton Dickinson Immunocytometry System, San Jose, CA, USA) was used to analyze the granulocyte expression. FlowJo® software (Tree Star, Inc., Ashland, OR, USA) was used to analyze the data.

### Histological analysis

Histological analysis was performed according to [21,22] modified. The left lung was prepared, cut into 5 µm and hematoxylin and eosin (H.E.) stained as previously described [23] modified. From each sample four representative photos were taken (magnification x200). Five high power fields were randomly assigned to each photo. Subsequently, alveolar wall thickness was analyzed by ImageJ software (ImageJ, U. S.National Institutes of Health, Bethesda, Maryland, USA). For each high power fields, a modified ALI score was used to determine the degree of lung damage: In brief, (a) thickness of the alveolar walls, (b) infiltration or aggregation of inflammatory cells, (c) hemorrhage and (d) alveolar congestion were rated in a blinded classification. Each sample was graded according to the point scale: 0: minimal damage, 1: mild damage, 2: moderate damage, 3: severe damage, 4: maximal damage. The samples were evaluated by two professional and the degree of ALI was assessed by the sum of scores, ranging from 0 to 16. The average of the sum of each reading was compared among groups.

### Cytokine and chemokine analysis

A BD^TM^ Cytometric Bead Array (CBA) Mouse Inflammation Kit (BD Biosciences, San Jose, USA) was used to measure the IL-6, IL-10, MCP-1, IFN-γ, TNF- α, and IL-12p70 in the animals’ BAL supernatant. The assays were performed according to the manufacturer’s instructions. 

### Protein analysis in the BAL

The BAL supernatant aliquots were placed in a 96-well ELISA plate (10 μL/well), and 250 μL of Bradford reagent (Sigma-Aldrich, St. Louis, USA) was added to each well; after a 30 min incubation period, the absorbance was measured at 595 nm. A standard curve using bovine serum albumin (BSA, Sigma-Aldrich, St. Louis, USA) was obtained by plotting the net absorbance vs. the protein concentration (1.500 - 100 µg/mL) to determine the protein concentration in the samples.

### Adhesion molecule expression

The recovered BAL cells were adjusted to 1 x 10^6^ cells and stained with APC-conjugated anti-mouse CD62L (L-selectin), clone MEL-14 (Biolegend, CA, USA); FITC-conjugated anti-mouse CD18 (beta2-integrin), clone M18/2 (eBioscience, CA, USA); and PE-conjugated anti-mouse CD31 (PECAM), clone 390 (Biolegend, CA, USA), according to the manufacturer’s instructions. A flow cytometer (FACSCalibur, Becton Dickinson Immunocytometry System, San Jose, CA, USA) was used to analyze the molecular cell surface expression. FlowJo® software (Tree Star, Inc., Ashland, OR, USA) was used to analyze the data.

### Statistical analysis

GraphPad Prism version 5.0 (GraphPad Software, Inc.) was used for statistical analysis. Parametric data were analyzed by one-way ANOVA followed by a Tukey-Kramer test for multiple comparisons. Non-parametric data were analyzed by a Mann Whitney U test for two-group comparisons or by a Kruskal-Wallis followed by Dunn´s tests for multiple comparisons. The BAL protein concentration data were first analyzed by normalizing to the percentage of control (vehicle + saline) and were then compared to the C2 (vehicle + LPS) and the E2 (JZL184+LPS) groups using the non-parametric tests. In all experiments, p ≤ 0.05 was considered significant. Data are presented as the mean ± S.D.

## Results

In both experiments, no differences were found in mice that received intranasal saline, whether they were treated (E1) or not (C1) with JZL184 (data not shown), i.e., JZL184 induced no effects in the absence of LPS-induced ALI.

### Treatment with JZL184 decreased leukocyte migration

#### BAL

**Figure 1:**
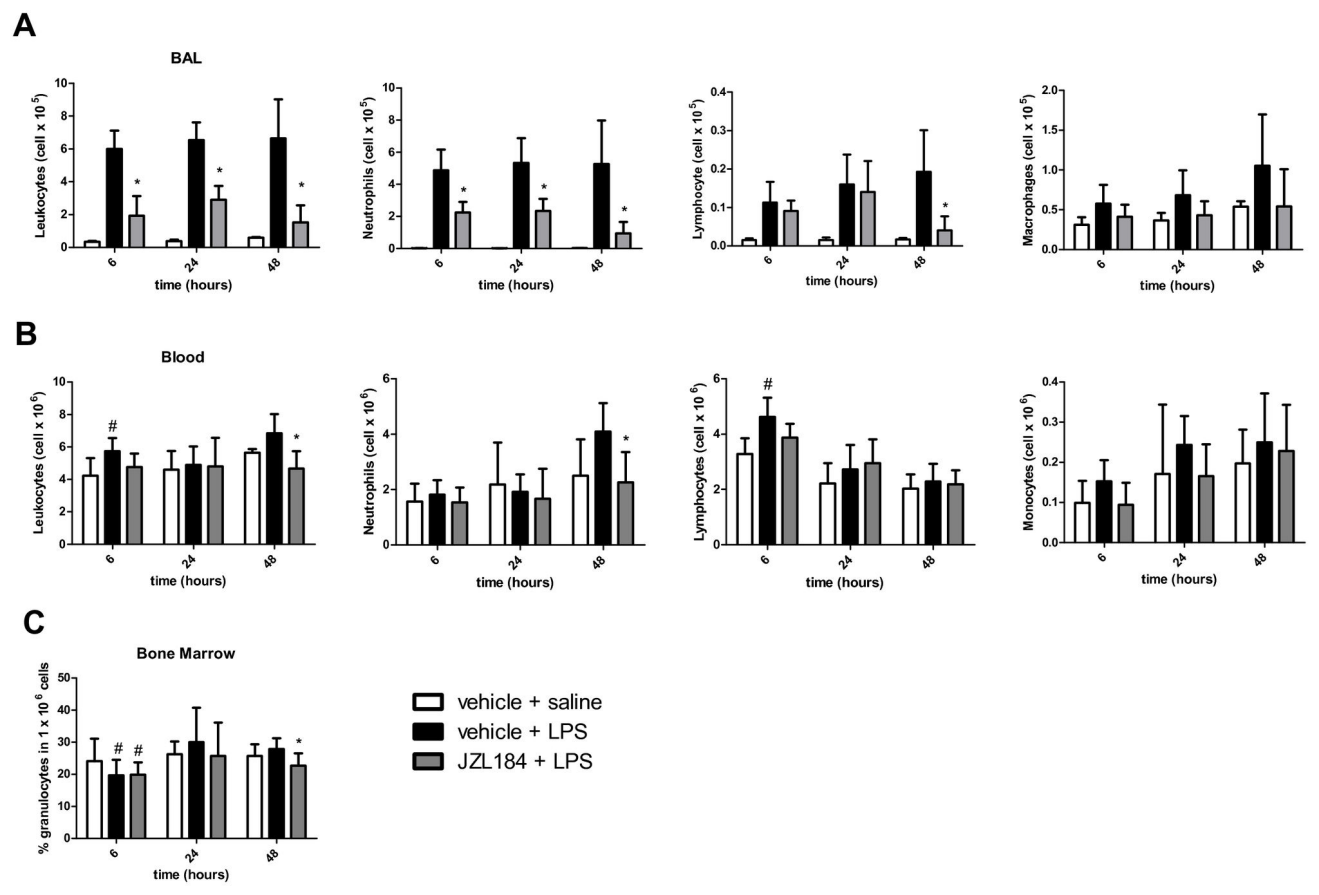
JZL184 effects in leukocyte migration into the lungs. (A) Total and differential leukocyte count in the bronchoalveolar lavage fluid, (B) Total and differential leukocyte count in the blood and (C) Percentage of granulocytes in the bone marrow. Data are presented as mean ± S.D., n=5-9 mice/group. *p<0.05, in relation to C2 group and #p<0.05 in relation to C1 group. One-way ANOVA and Tukey-Kramer tests.

First, we investigated the effects of MAGL inhibition (JZL184 16 mg/kg) on leukocyte migration into the murine lungs at 6, 24 and 48 hours after the LPS-induced ALI. It is noteworthy that the LPS instillation was effective in inducing lung inflammation, as observed by the number of leukocytes present in the BAL, 6 (F (2,17) = 48.16; p < 0.0001), 24 (F (2,17) = 49.44; p < 0.0001) and 48 (F (2,18) = 23.19; p < 0.0001) hours after the LPS nasal instillation (Figure 1A). Treatment with JZL184 decreased the leukocyte counts in the BAL at 6 (F (2,17) = 48.16; p < 0.0001), 24 (F (2,17) = 49.44; p < 0.0001) and 48 (F (2,18) = 23.19; p < 0.0001) hours after LPS-induced ALI (Figure 1A). 

Differential analysis of the leukocytes found in the BAL of JZL184-treated mice showed that treatment decreased the neutrophil counts at 6 (F (2,17) = 46.02; p < 0.0001), 24 (F (2,17) = 37.04; p < 0.0001) and 48 (F (2,18) = 14.70; p < 0.0001) hours after LPS-induced ALI, as well as the lymphocyte counts at 48 hours (F (2,18) = 9.926; p < 0.001) after LPS instillation (Figure 1A). No differences were found in the macrophage count in the BAL taken at 6, 24 and 48 hours after LPS nasal instillation (Figure 1A).

#### Blood

The effects of MAGL inhibition, by 16 mg/kg JZL184, were also analyzed in the blood at 6, 24 and 48 hours after LPS-induced ALI. An increase in leukocyte count (F (2,17) = 4.294; p < 0.05), mainly of lymphocytes (F (2,17) = 6.923; p < 0.001), was evident in the blood of the C2 group at 6 hours after LPS instillation (Figure 1B); at this moment, this increase was not abrogated by the JZL184 treatment (Figure 1B). However, in relation to data taken in the C2 group, the JZL184 treatment decreased the leukocyte (F (2,18) = 8.544; p < 0.001) and neutrophil (F (2,18) = 6.514; p < 0.001) counts in the blood 48 hours after the ALI induction (Figure 1B). 

No differences were found for the monocyte counts in the blood in all periods evaluated (Figure 1B), as well as for the total and differential leukocyte counts in the blood taken 24 hours after the LPS intranasal instillation (Figure 1B).

#### Bone marrow

The mice in the C2 group exhibited a decreased expression of granulocytes in the bone marrow 6 hours after the LPS-induced ALI (F (2,17) = 6.212; p < 0.05) (Figure 1C); this finding was not abrogated by the JZL184 treatment as observed in the mice of the E2 group (F (2,17) = 6.212; p < 0.05) (Figure 1C). Further analysis showed that 48 hours after the LPS intranasal instillation, the expression of granulocytes in the bone marrow of the mice of the E2 group was smaller than the expression measured in the C2 group (F (2,17) = 3.553; p < 0.05) (Figure 1C). However, no differences were found in the granulocyte expression in the bone marrow 24 hours after LPS intranasal instillation (Figure 1C).

### Treatment with JZL184 decreased lung damage

**Figure 2:**
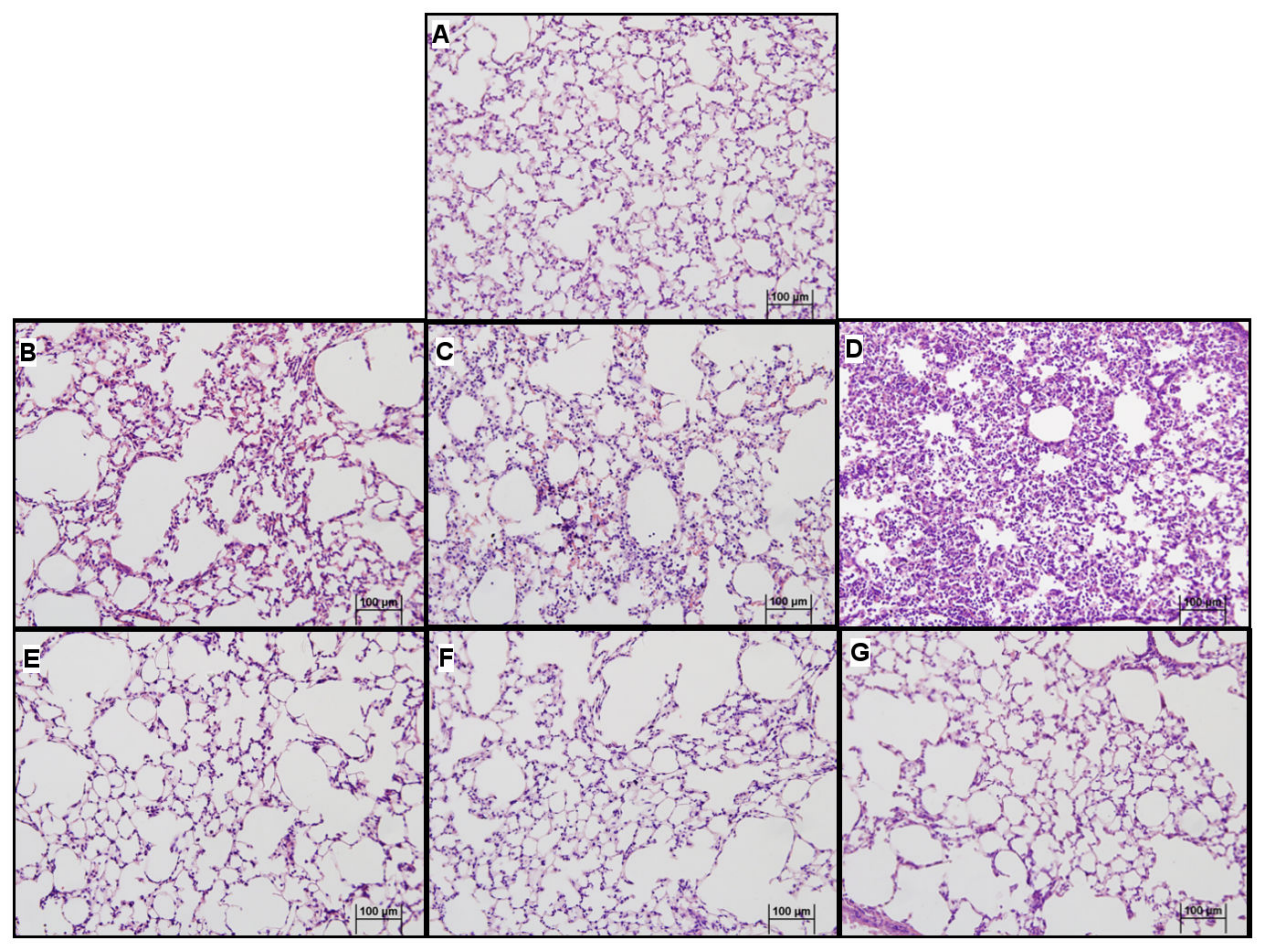
JZL184 effects on lung architecture in LPS-induced lung damage. (A) Non inflamed control; (B) Inflamed control 6 hours, (C) Inflamed control 24 hours and (D) Inflamed control 48 hours after LPS intranasal instillation; (E) JZL184 treated animals 6 hours, (F) JZL184 treated animals 24 hours and (G) JZL184 treated animals 48 hours after LPS intranasal instillation. Sections from the left lung lobe were stained with hematoxylin and eosin. Representative pictures are shown for each experimental group (magnification = 200X).

**Figure 3:**
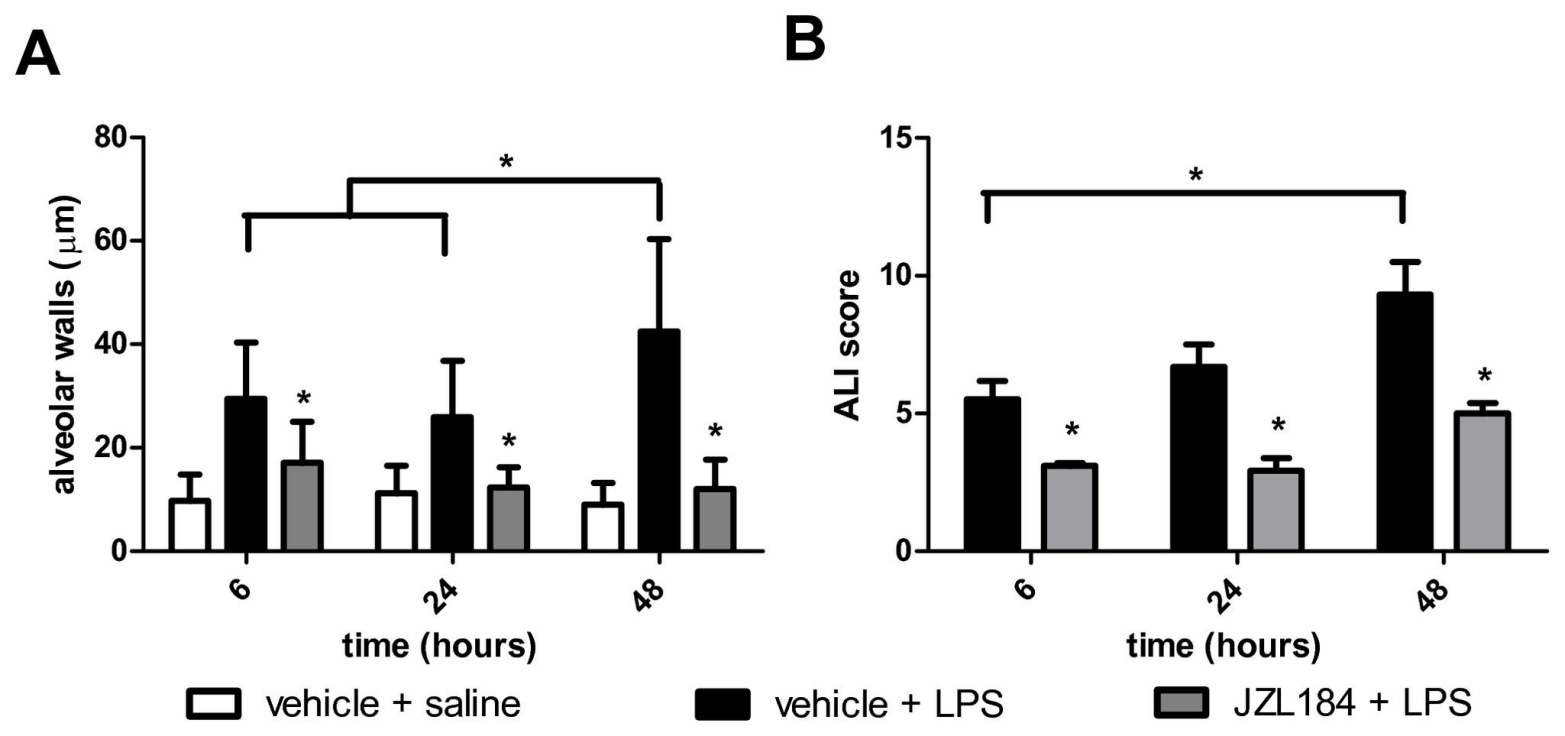
JZL184 effects LPS-induced lung damage. (A) Alveolar wall thickness and (B) ALI score. Data are presented as mean ± S.D., n=5-6 mice/group. *p<0.05, in relation to C2 group. (A) One-way ANOVA and Tukey-Kramer tests; (B) Mann Whitney U test for two group comparison.

In the presented model of acute lung injury, H.E. staining of lung sections showed that, as compared to control condition (Figure 2A), LPS treatment clearly stimulated the formation of hemorrhage, alveolar congestion and, mainly, increase in thickness of the alveolar walls and infiltration or aggregation of inflammatory cells (Figure 2B-D). These changes were found more pronounced over time (Figure 2B-D). In sharp contrast, the JZL184 treatment reduced LPS-induced lung damage in all evaluated periods, reflected mainly by the prevent an increase in thickness of the alveolar walls and infiltration or aggregation of inflammatory cells (Figure 2E-G). These findings were confirmed by quantitative analyse and by ALI score: JZL184 treatment prevented alveolar wall thickening (F (1,11) = 130.3; p < 0.0001 6 hours; F (1,10) = 105.7; p < 0.0001 24 hours and F (1,10) = 220.7; p < 0.0001 48 hours after LPS intranasal instilation) (Figure 3A) and prevented further damage tissue (U = 8.0; p < 0.001 6 hours; U = 8.0; p < 0.001 24 hours and U = 5.0; p < 0.05 48 hours after LPS intranasal instilation) (Figure 3B), respectively.

### Treatment with JZL184 changed the adhesion molecule expression in the neutrophils of the bronchoalveolar lavage fluid and blood

**Figure 4:**
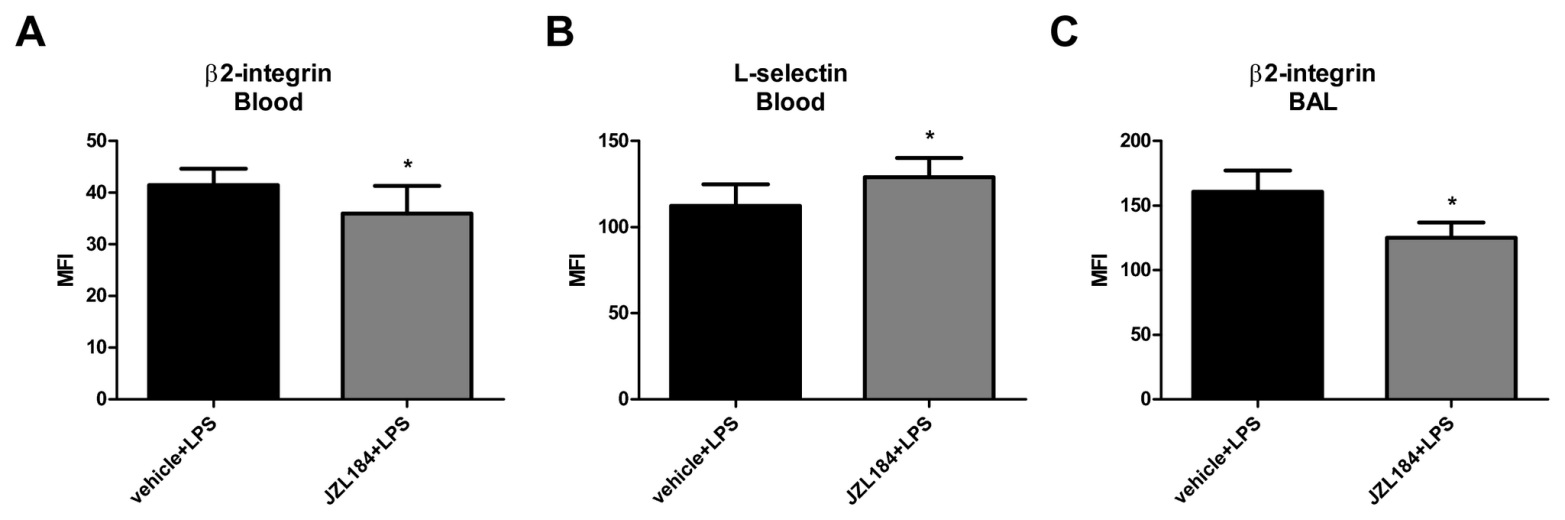
JZL184 effects in adhesion molecules expression in LPS-induced ALI. (A) β2-integrin and (B) L-selectin in the blood 6 hours after LPS-induced ALI. (C) β2-integrin in the bronchoalveolar lavage fluid 48 hours after LPS-induced ALI. Data are mean ± S.D., n=5-9 mice/group. *p<0.05 in relation to C2 group. Mann Whitney U test for two group comparison.

We next investigated the expression of the adhesion molecules (L-selectin, β2-integrin and PECAM) in the leukocytes recovered from the blood and the BAL of mice treated or not with JZL184. In relation to the animals of the C2 group, the JZL184 treatment decreased the β2-integrin expression (U = 8.0; p < 0.05) (Figure 4A); however, at the same time, this treatment increased the L-selectin expression (U = 9.0; p < 0.05) (Figure 4B) in the neutrophils taken from the blood 6 hours after LPS-induced ALI. No differences were found for PECAM expression in the neutrophils of the blood taken in all periods analyzed (data not shown) or in β2-integrin and L-selectin expression at 24 and 48 hours after LPS intranasal instillation (Table 1). Furthermore, it was also observed that MAGL inhibition by JZL184 decreased the β2-integrin expression (U = 7.0; p < 0.001) in neutrophils in the BAL 48 hours after LPS-induced ALI (Figure 4C). However, significant difference were not found for both L-selectin (Table 1) and PECAM (data not shown) expression in the neutrophils taken from BAL in all of the periods analyzed.

**Table 1:** Adhesion molecules expression in the BAL and blood of mice 6, 24 and/or 48 hours after LPS-induced acute lung injury.

**Analyzed material**	**Adhesion molecule**	**Time after LPS instillation**	**Vehicle + LPS**	**JZL184 + LPS**
**BAL**	Β2-integrin	6 hours	110.3±3.724	107.1±4.638
	L-selectin	6 hours	32.74±4.794	32.54±4.104
	Β2-integrin	24 hours	66.34±4.885	60.40±6.251
	L-selectin	24 hours	19.40±0.9345	18.40±1.578
	L-selectin	48 hours	17.50±1.442	18.05±1.943
**Blood**	Β2-integrin	24 hours	40.50±2.606	42.35±4.270
	L-selectin	24 hours	19.70±1.457	19.00±3.616
	Β2-integrin	48 hours	81.57±9.448	79.90±7.754
	L-selectin	48 hours	47.29±6.498	46.91±3.798

Data are mean ± S.D., n=5-9 mice/group. Mann Whitney U test for two groups comparisons.

### Treatment with JZL184 decreased vascular permeability

**Figure 5:**
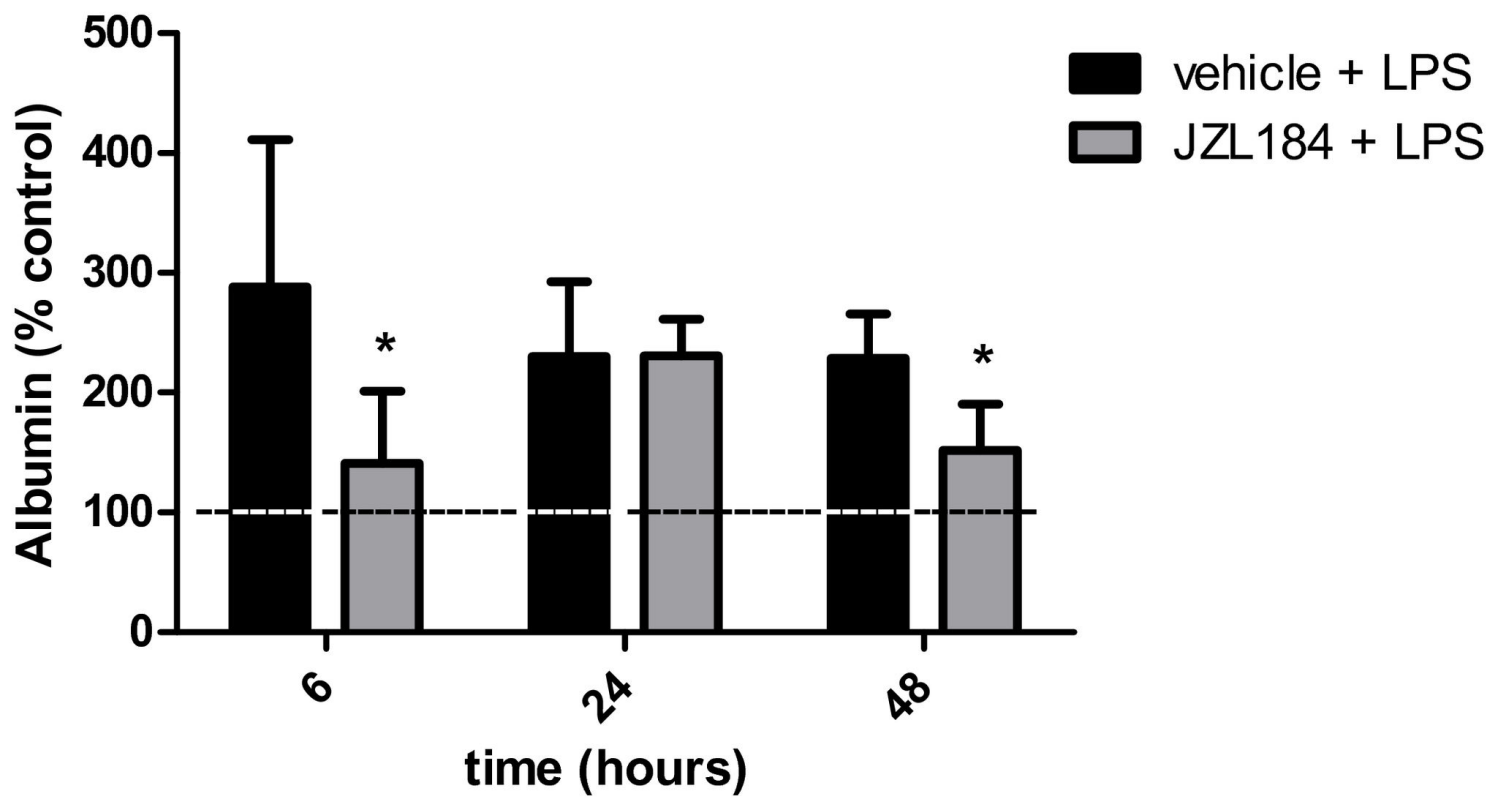
JZL184 effects on albumin concentration in the bronchoalveolar lavage fluid. Data are mean ± S.D., n=5-8 mice/group. *p<0.05 in relation to C2 group. Mann Whitney U test for two groups comparisons. Data are normalized to protein of control.

Vascular permeability within the lungs was measured by the albumin concentration in the BAL. It was shown that JZL184 decreased the protein concentration in the BAL in relation to mice in the C2 group at 6 (U = 3.0; p < 0.05) and 48 (U = 4.0; p < 0.001) hours after LPS-induced ALI (Figure 5). 

### Treatment with JZL184 decreased the production of pro-inflammatory cytokines/chemokines in the bronchoalveolar lavage fluid

**Figure 6:**
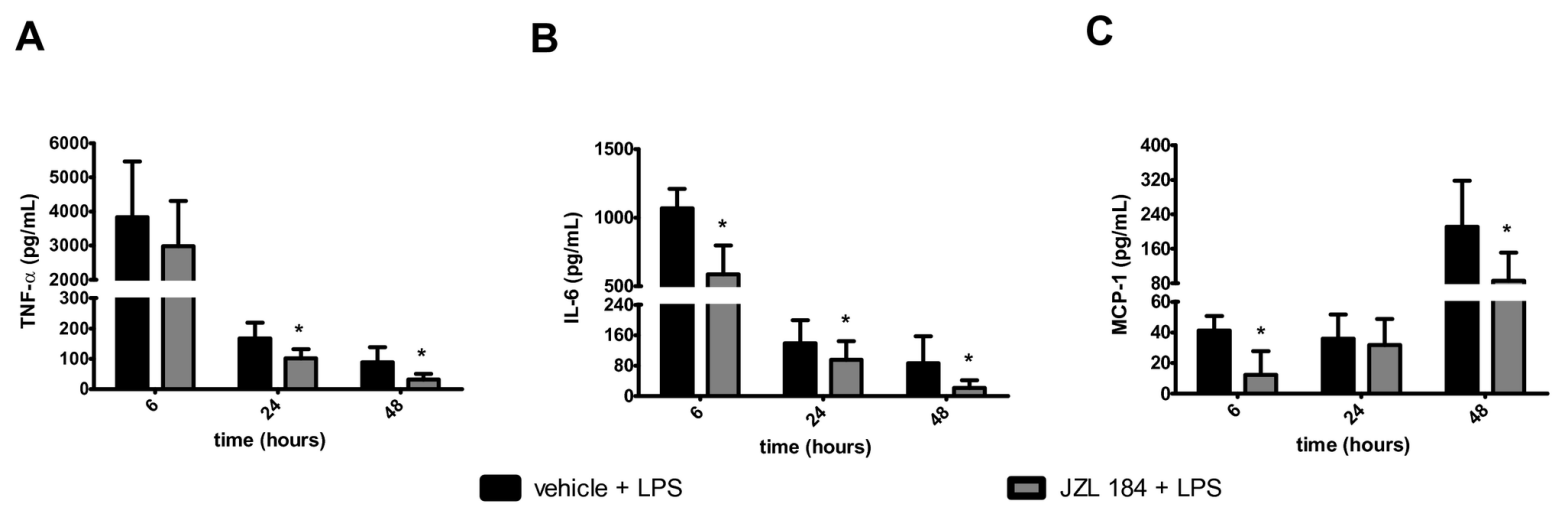
JZL184 effects in cytokines/chemokines concentration in the bronchoalveolar lavage fluid. (A) TNF-α, (B) IL-6 and (C) MCP-1. Data are mean ± S.D., n=5-8 mice/group. *p<0.05 in relation to C2 group. Mann Whitney U test for two groups comparisons.

In relation to mice in the C1 group, large increases in the TNF- α and IL-6 concentrations were observed in the C2 group 6 hours after the LPS-induced ALI (Figure 6A-B). Gradual reductions in the TNF- α and IL-6 concentrations were detected at 24 and 48 hours after the LPS intranasal instillation (Figure 6A-B). However, at the same time, a gradual increase in the MCP-1 concentration was observed in the BAL in animals of the C2 group in all of the evaluated periods (Figure 6C). The JZL184 treatment decreased the TNF-α concentration at 24 (U = 3.0; p < 0.05) and 48 (U = 4.0; p < 0.001) hours after LPS-induced ALI (Figure 6A), as well as the IL-6 concentration at 6 (U = 2.0; p < 0.05), 24 (U = 6.0; p < 0.05) and 48 (U = 9.0; p < 0.05) hours after LPS-induced ALI (Figure 6B). Further analysis showed that JZL184 treatment (E2 group) also exhibited a reduced MCP-1 concentration 6 (U = 4.0; p < 0.001) and 48 (U = 7.0; p < 0.05) hours after LPS-induced ALI (Figure 6C). Statistically significant differences were not observed among the groups for the IL-10, IFN-γ and IL-12p70 concentrations measured in the BAL of mice taken at 6, 24 and 48 hours after the LPS intranasal instillation (Table 2).

**Table 2:** Cytokines and chemokines concentrations in the BAL 6, 24 and/or 48 hours after LPS-induced acute lung injury.

**Cytokines / Chemokines**	**Time after LPS instillation**	**Vehicle + LPS (pg/mL)**	**JZL184 + LPS (pg/mL)**
TNF-α	6 hours	3836±1631	2984±1329
IL-10	6 hours	1.492±3.654	2.690±4.624
IFN-ɣ	6 hours	1.150±0.6314	0.3386±0.5786
IL-12p70	6 hours	0.0±0.0	0.0±0.0
MCP-1	24 hours	35.91±15.82	37.01±10.95
IL-10	24 hours	7.667±5.777	8.683±4.226
IFN-ɣ	24 hours	0.3143±0.5383	0.6200±0.5803
IL-12p70	24 hours	0.0±0.0	0.0±0.0
IL-10	48 hours	4.337±5.504	6.349±5.340
IFN-ɣ	48 hours	1.984±2.133	1.135±0.7783
IL-12p70	48 hours	0.0±0.0	0.0±0.0

Data are mean ± S.D. Mann Whitney U test for two groups comparisons.

### CB_1_ and CB_2_ receptor antagonism attenuated the anti-inflammatory effects induced by JZL184 on ALI

**Figure 7:**
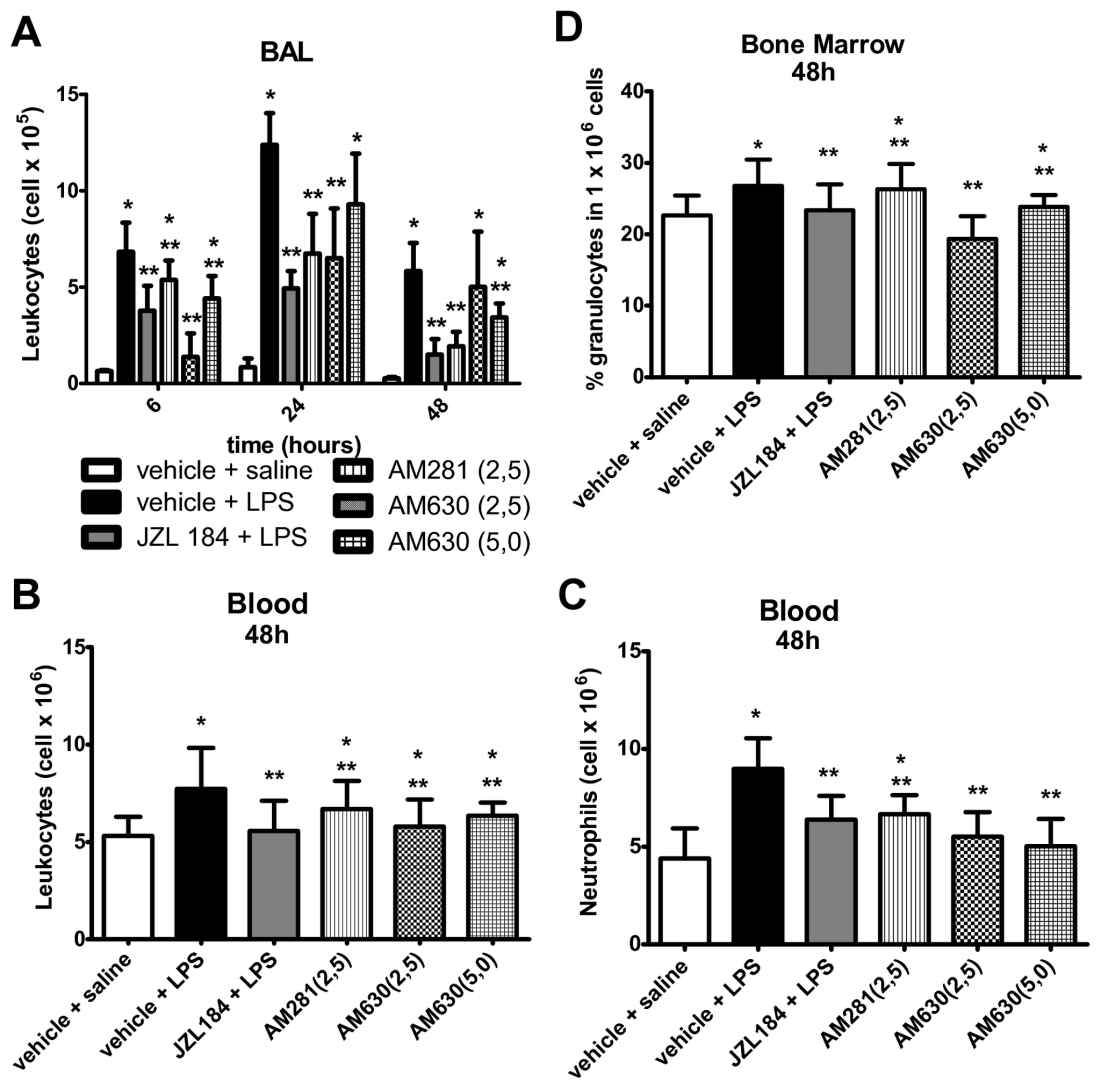
CB1 and CB2 receptors participation in leukocyte migration into the lungs. (A) Leukocyte count in the bronchoalveolar lavage fluid, (B) Leukocyte count in the blood, (C) neutrophils count in the blood and (D) the percentage of granulocytes in the bone marrow. Data are mean ± S.D., n=5-8 mice/group. * and **p<0.05. One-way ANOVA and Tukey-Kramer tests.

The roles of the CB_1_ and CB_2_ cannabinoid receptors in the anti-inflammatory effects of JZL184 were next analyzed using AM281 (a CB_1_ receptor antagonist) and AM630 (a CB_2_ receptor antagonist) as pharmacological tools. The data showed that the AM281 and AM630 treatments partially abrogated the JZL184-induced actions on leukocyte migration into the lungs 6 hours after LPS instillation (F (5,28) = 13.53) (Figure 7A). However, only the AM630 treatment abrogated the JZL184-induced inhibition of leukocyte migration into the lungs at 24 (F (5,28) = 23.33; p < 0.05) and 48 (F (5,25) = 13.73; p < 0.001) hours after LPS intranasal instillation (Figure 7A). Further analysis revealed that the AM281 and AM630 treatments partially reverted both the JZL184-induced actions on the leukocyte count in the blood (F (5,25) = 3.61) (Figure 7B) and the percentage of granulocytes in the bone marrow (F (5,25) = 6.336) (Figure 7D) at 48 hours after LPS-induced ALI. It was also shown that the JZL184-induced actions on the neutrophil count in the blood were partially reversed by the AM281 treatment (F (5,25) = 7.498) (Figure 7C).

**Figure 8:**
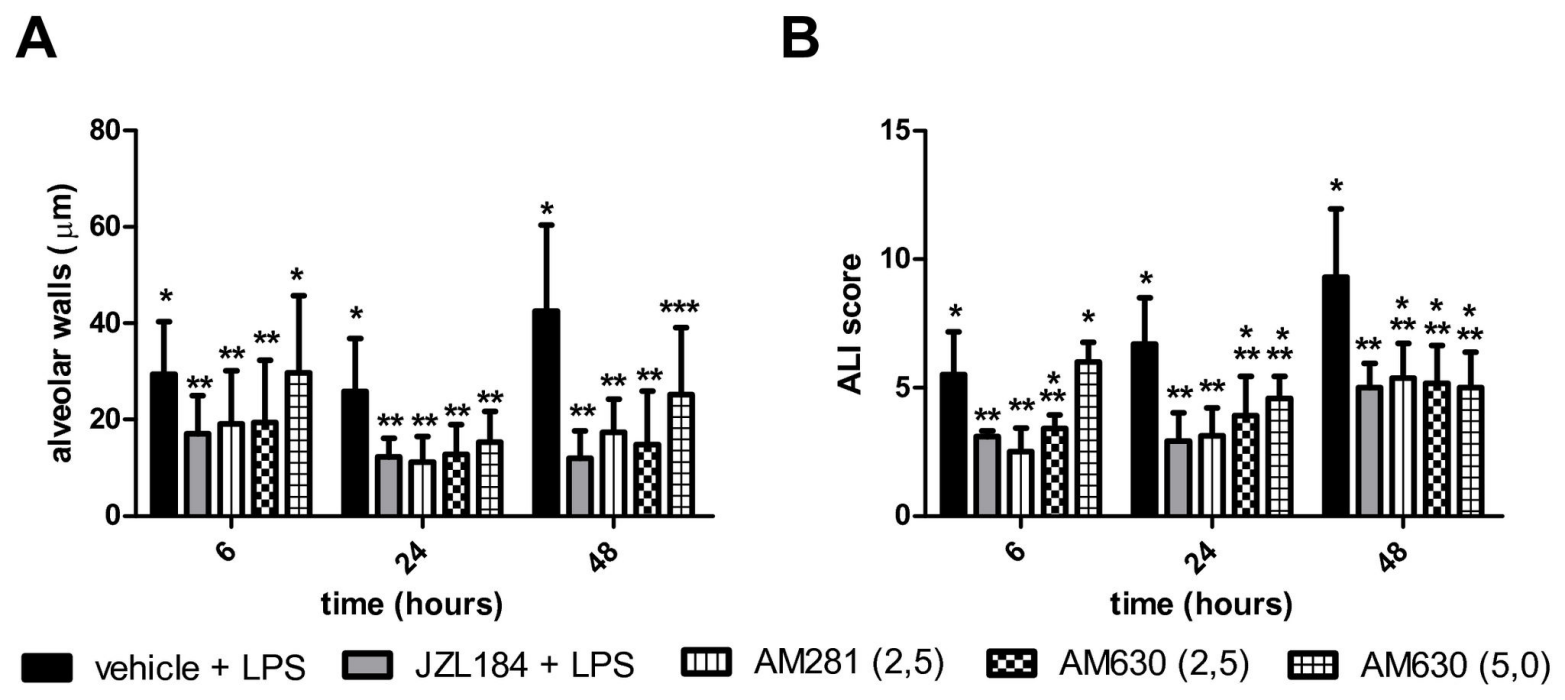
CB1 and CB2 receptors participation in LPS-induced lung damage. (A) Alveolar wall thickness and (B) ALI score. Data are presented as mean ± S.D., n=5-8 mice/group. * and **p<0.05. (A) One-way ANOVA and Tukey-Kramer tests; (B) Kruskal-Wallis and Dunn´s tests for multiples comparisons.

The JZL184 effects in prevented alveolar wall tickening and the lung damage was reversed with AM630 (5mg/kg) treatment 6 hours after LPS intranasal instillation (F (5,29) = 61.23; p < 0.0001 and KW = 21.44; p < 0.05) (Figure 8A-B).

**Figure 9:**
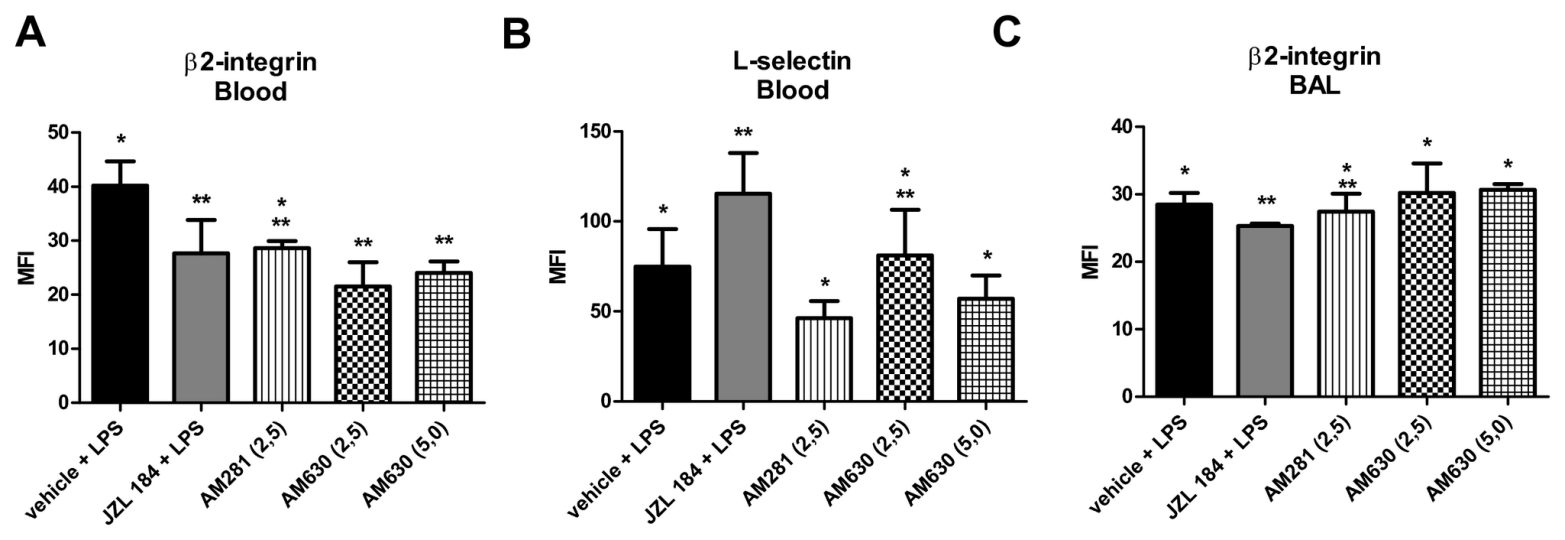
CB1 and CB2 receptors participation in adhesion molecules expression in LPS-induced ALI. (A) β2-integrin in the blood 6 hours after LPS-induced ALI, (B) L-selectin in the blood 6 hours after LPS-induced ALI and (C) β2-integrin in the bronchoalveolar lavage fluid 48 hours after LPS-induced ALI. Data are mean ± S.D., n=5-8 mice/group. * and **p<0.05. Kruskal-Wallis and Dunn´s tests for multiples comparisons.

Further analysis showed that the JZL184-induced actions on the expression of adhesion molecules in the blood and in the BAL were reduced or abrogated by the use of both AM281 and AM630. Thus, AM281 reduced the JZL184-induced inhibition of β2-integrin expression in the blood 6 hours after ALI (KW = 20.30) (Figure 9A). Furthermore, AM630 and AM281 reduced the JZL184-induced increase of L-selectin expression (KW = 24.65) (Figure 9B) in the blood 6 hours after LPS-induced ALI. The JZL184-induced decrease in the β2-integrin expression in the BAL was also reduced by AM281 (KW = 20.39) and reverted by AM630 (KW = 20.39; p<0.0001 for 5.0 mg/kg dose and KW = 20.39; p<0.05 for 2.5 mg/kg dose) treatments (Figure 9C).

**Figure 10:**
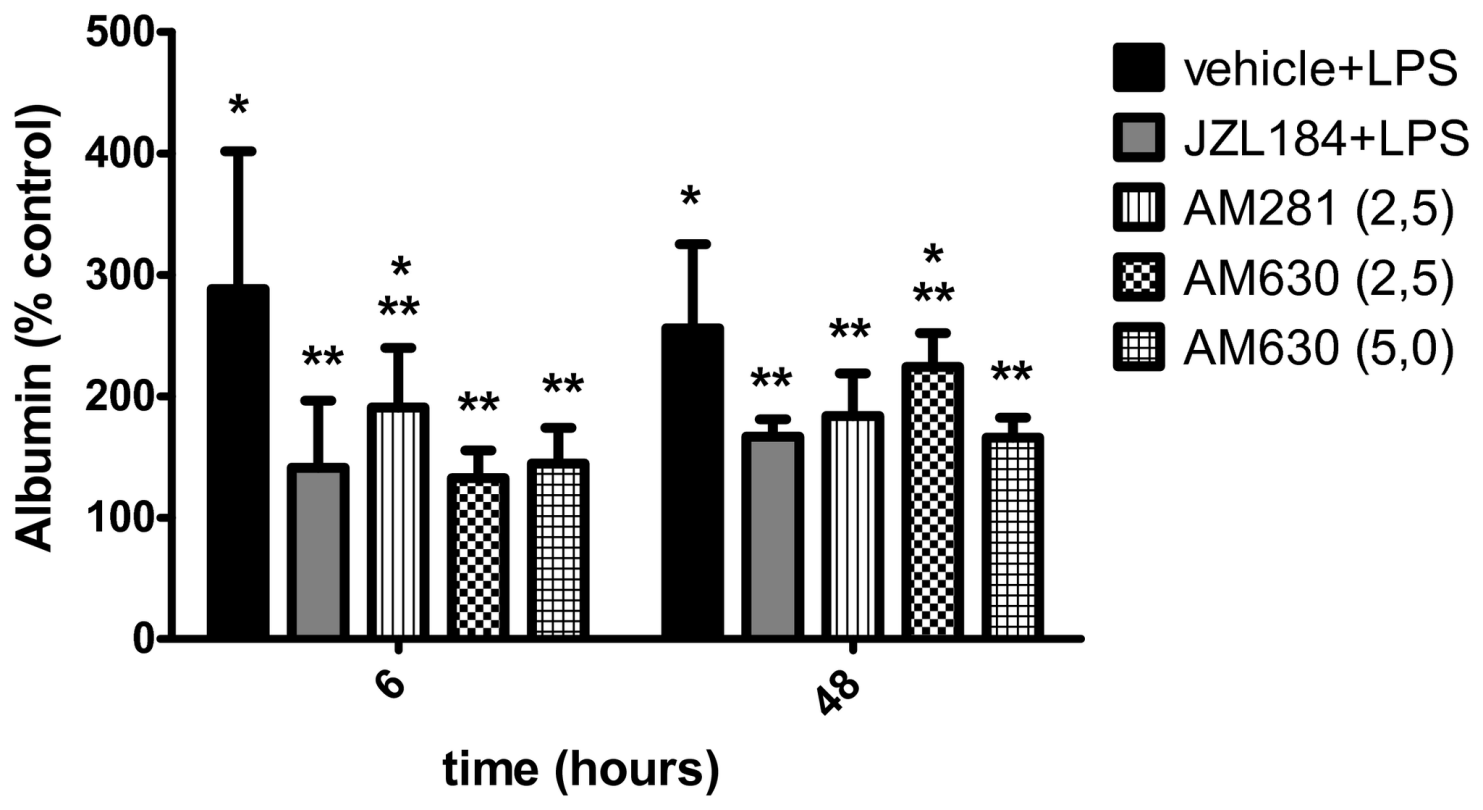
CB1 and CB2 receptors participation on albumin concentration in the bronchoalveolar lavage fluid. Data are mean ± S.D., n=5-8 mice/group. * and **p<0.05. Kruskal-Wallis and Dunn´s tests for multiples comparisons.

As depicted in Figure 10, the AM281 (KW = 21.04) and the AM630 (KW = 13.84) treatments attenuated the JZL184-induced effects on the lungs’ vascular permeability at 6 and 48 hours after LPS-induced ALI, respectively. 

**Figure 11:**
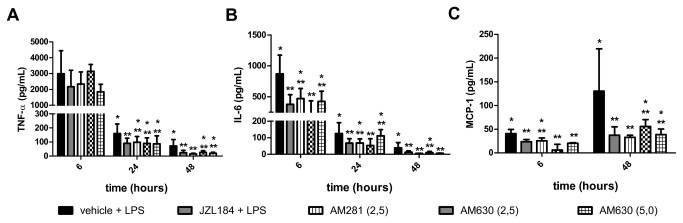
CB1 and CB2 receptors participation in cytokine/chemokine concentrations in the bronchoalveolar lavage fluid. (A) TNF-α, (B) IL-6 and (C) MCP-1. Data are mean ± S.D., n=5-8 mice/group. * and **p<0.05. Kruskal-Wallis and Dunn´s tests for multiples comparisons.

Finally, it was observed that both the AM281 and/or AM630 treatments attenuated the JZL184-induced actions on the following pro-inflammatory cytokine/chemokine concentrations measured in the BAL 6, 24 or 48 hours after LPS-induced ALI: TNF- α (KW = 13.33, 24 hours and KW = 21.05, 48 hours after LPS instillation) (Figure 11A), IL-6 (KW = 13.27, 6 hours, KW = 19.80, 24 hours and KW = 22.02, 48 hours after LPS instillation) (Figure 11B) and MCP-1 (KW = 20.43, 6 hours and KW = 19.10, 48 hours after LPS instillation) (Figure 11C).

## Discussion

JZL184 is commonly known as a highly efficacious and selective inhibitor of MAGL, a 2AG-degrading enzyme [7]; it has been reported that JZL184 has potent immunosuppressive and anti-inflammatory properties [9]. We are now reporting that a single dose of JZL184 (16 mg/kg, i.p.) is able to decrease several lung inflammation parameters during the course of a murine model of LPS-induced ALI, specifically leukocyte migration (neutrophils, lymphocytes and macrophages) to the lungs, adhesion molecule expression in the BAL and blood, vascular permeability and cytokine/ chemokine production in the BAL. We also showed that signaling through the CB_1_ and CB_2_ cannabinoid receptors might have a relevant role in the anti-inflammatory effects now being reported for JZL184.

Organ dysfunction and failure from inflammatory response continues to be the major problem after injury in many clinical conditions such as sepsis, acute pancreatitis and hemorrhagic shock [24]. In humans, the acute lung injury (ALI) that manifests clinically as acute respiratory distress syndrome (ARDS), a major component of multiple organ dysfunction syndrome, is described as “syndrome of inflammation and increased capillary endothelial permeability” [25,26]. Inflammatory mediators play a key role in the pathogenesis of ARDS, which is the primary cause of death in these conditions [24].

Neutrophil influx into the interstitium and the bronchoalveolar space is considered a keystone for the progression of ALI [12]. We found that JZL184 treatment decreased total leukocyte migration into the lungs of mice at 6, 24 and 48 hours after LPS-induced ALI, as shown by the BAL leukocyte count and ALI score. Specifically, we observed that the neutrophils and the lymphocytes were greatly reduced in the BAL taken from JZL184-treated mice in relation to the inflamed non-treated mice. Leukocyte migration into the site of inflammation is known to be important because the presence of high amounts of inflammatory cells, especially neutrophils, might damage the lung tissue [27]. Thus, it appears feasible to suggest that a reduction in the inflammatory process, induced by JZL184 in mice, could decrease ALI lung damage.

In the presence of an inflammatory process, the blood cells are immediately recruited to the site of inflammation; the bone marrow produces the largest quantity of leukocytes that migrate to the bloodstream and are directed to the inflammatory focus [28].

The control mice treated with LPS exhibited an increased number of lymphocytes in their blood 6 hours after the LPS instillation; the JZL184-treated animals exhibited a decrease in this neutrophil number in the blood 48 hours after LPS-induced ALI. It is known that the inflammatory process begins at the microcirculation site, specifically by the formation of the intercellular spaces that are responsible for the increased microvascular permeability observed during the course of ALI [29]. Thus, it appears that a reduction in the epithelial cell barrier function might have facilitated the influx of a fluid rich in cells and macromolecules into the lung alveolar space. Indeed, we showed that MAGL inhibition by JZL184 reduced the vascular permeability to protein influx into the BAL at 6 and 48 hours after LPS instillation, a fact that might have contributed to the observed decrease in lung inflammation. In our experimental model, JZL184 also induced a decrease in the number of granulocytes produced in the bone marrow in relation to the inflamed control lung. 

Neutrophil migration into the lungs is not sufficient to cause ALI; neutrophil activation and pro-inflamatory cytokine release are also required [12]. To initiate the inflammatory process, the immune system cells produce and release cytokines and chemokines to recruit inflammatory cells to the site of inflammation. 

 TNF-α is released during the first 30–90 min after exposure to LPS and in turn activate a second level of inflammatory cascades including cytokines, lipid mediators, and reactive oxygen species, as well as up-regulating cell adhesion molecules that result in the initiation of inflammatory cell migration into tissues [30]. TNF-α is present in the bronchoalveolar lavage fluid of patients at risk for ARDS and with established ARDS [31]. Interleukin 6 (IL-6) is produced by a wide range of cells including monocytes/macrophages, endothelial cells, fibroblasts, and smooth muscle cells in response to stimulation by endotoxin, IL-1β, and TNF-α [30,32–34]. Circulating levels of IL-6 have been shown to be excellent predictors of the severity of ARDS of different aetiologies such as sepsis [35] and acute pancreatitis [36]. Chemokines can be classified as constitutive (developmentally regulated) or inducible (inflammatory). Chemokines have chemotactic and activating effects on leukocyte subsets, which provide a key stimulus for directing leukocytes to areas of injury [24].

Treatment with JZL184 has been reported to block cytokine expression in the gut or blood in a gastric inflammation model [9][10]. A reduction in pro-inflammatory cytokine (TNF-α and IL-6) and chemokine (MCP-1) production in JZL184-treated mice in relation to the inflamed control group was presently observed. These results appear to be extremely important because cytokines and chemokines are essential for both inflammation establishment and phagocyte activation. This action might explain the presently observed reduction in leukocyte migration into the lungs. MCP-1, also known as CCL2, is a molecule that attracts monocytes and lymphocytes as well as neutrophils to the site of inflammation [37]. Thus, it is reasonable to suggest that JZL184, by reducing MCP-1 in the BAL, decreased the total leukocyte count in the BAL. 

Our data suggest that the inhibition of adhesion molecules by JZL184 might have been involved in the anti-inflammatory effects presently being reported. Leukocyte migration into inflamed tissues involves complex interactions of leukocytes with the endothelium through the regulated expression of surface adhesion molecules. In the present study, we showed that JZL184 treatment (1) decreased the cellular expression of β2-integrin in the blood 6 hours after the LPS-induced ALI (2), increased the cellular expression of L-selectin in the blood 6 hours after the LPS instillation, and (3) decreased the cellular expression of β2-integrin in the BAL 48 hours after the induction of inflammation. Together, these facts appear to justify the less intense inflammatory response observed in the mice of the JZL-treated group in relation to those of the inflamed control group. Indeed, β2-integrin and L-selectin are known to increase the leukocyte adhesion process [38][39]. Time course studies of the β2-integrin and L-selectin expression during inflammation have shown an inverse relationship in the serum, where β2-integrins are upregulated and L-selectins are downregulated [38][39]. It has been speculated that this inverse relationship is necessary to allow the adhesion of the neutrophils [40]. Taking this fact into account and also the changes observed here in the adhesion molecule expression, it seems possible to suggest that adhesion molecules might have accounted for the reduction in inflammatory cell adhesion in the JZL184-treated mice in the present experiment.

JZL184 is known to exert its effects by inhibiting MAGL, thereby increasing the levels of the endocannabinoid 2-AG [7]. 2-AG and anandamide are signaling lipids that bind to the cannabinoid receptors CB_1_ [1] and CB_2_ [3]. Activation of the CB_1_ and CB_2_ receptors has been shown to play an important role in inflammation [9]. Endogenous and exogenous cannabinoid receptor agonists are involved in leukocyte activation and cytokine production in different inflammatory conditions [41–46].  The 2-AG, mainly through the CB2 receptor, stimulates inflammatory reactions and allergic responses by inducing robust adhesion of leukocytes to vascular endothelial cells [47]. In addition, other non-hematopoietic cells, such as endothelial cells, can respond to ligands of CB receptors. The CB1 and CB2 receptors are expressed during inflammatory angiogenesis; CB1 receptor appeared to be strongly involved in leukocyte accumulation and cytokine production, CB2 receptor is mainly linked to a pro-angiogenic response, especially through early neutrophil activation. Double blockade of cannabinoid receptors favored slightly better inhibition of inflammatory angiogenesis [48]. In the skin samples of healthy dogs, CB1 and CB2 receptors immunoreactivity was detected in various types of cells in the epidermis and in cells in the dermis, including perivascular cells with mast cell morphology, fibroblasts, and endothelial cells; in the skin samples of dogs with atopic dermatitis, CB1 and CB2 immunoreactivity was stronger than it was in skin samples of healthy dogs [49].

Therefore, to evaluate the involvement of the CB_1_ and CB_2_ receptor activation in the JZL184-induced anti-inflammatory effects, we used the CB_1_ (AM281) and CB_2_ (AM630) antagonists before the JZL184 treatment. Remarkably, the AM281 or AM630 treatments reduced all of the anti-inflammatory effects of JZL184 reported here, i.e., leukocyte migration into the lungs (from bone marrow to the blood and from the blood to the lungs), the lung damage, adhesion molecule expression, vascular permeability reduction and the production of pro-inflammatory cytokines/chemokines at 6, 24 or 48 hours after LPS-induced ALI. It has been reported that CB_1_ and, mainly, CB_2_ receptor activation decreased the inflammatory response during drug-induced gastric hemorrhages [10], as well as in models of colitis, a finding also observed during a systemic-induced inflammation in mice [9]. Furthermore, neutrophil migration in humans [50], cytokine/chemokine production in mice [9], vascular permeability in guinea pigs [51] and adhesion molecule expression in mice [52] have also been reported to be reduced by CB_1_ or CB_2_ activation. Thus, it appears feasible to suggest that the abrogation of the anti-inflammatory effects induced by JZL184 in LPS-induced ALI might have involved CB_1_ and CB_2_ receptor activation. However, it should not be forgotten that cannabinoid actions can be related to other mechanisms besides CB_1_ and CB_2_ activation, such as adenosine receptor activation [13] or partial FAAH inhibition by JZL184 administration [7]. Furthermore, beyond the CB_1_ and CB_2_ receptor participation, other possibilities might also explain the anti-inflammatory effects of JZL184 demonstrated in this work. One possibility may be the release of arachidonic acid from 2-AG and/or anandamide hydrolysis, a fact that would lead to eicosanoid (leukotriene) formation in neutrophils. It has been shown that 2-AG *in vitro* activates human neutrophils, an effect attributable to the leukotriene (LT) B4 ligation to specific neutrophil receptors (LTB4 receptors) [53]. 

Taken together, the present findings showed that a single dose of JZL184, given prior to LPS-induced ALI, resulted in an anti-inflammatory effect. It was also shown that the JZL184 effects on leukocyte migration from the blood to the lungs and from the bone marrow to the blood in the presently used model of ALI relies on adhesion molecule expression and on cytokine/chemokine participation. Finally, it was shown that the CB_1_ and CB_2_ cannabinoid receptors appear to be the most likely mechanism involved in the JZL184 anti-inflammatory effects in the LPS-induced ALI model. Although care should be taken when extrapolating the present data to patients. MAGL inhibition might become, in the future, a useful therapeutic tool for the treatment of inflammatory lung diseases, such as ALI and acute respiratory distress syndrome. 
